# The Process of Constructing a Health Tourism Destination Index

**DOI:** 10.3390/ijerph16224579

**Published:** 2019-11-19

**Authors:** Chia-Wen Lee, Ching Li

**Affiliations:** 1 College of Humanities and Communications, Yango University, Fuzhou 350015, China; lcwgg@mail2000.com.tw; 2 Graduate Institute of Sport, Leisure and Hospitality Management, National Taiwan Normal University, Taipei 106, Taiwan

**Keywords:** health tourism destination, analytic hierarchy process (AHP), health environment, health promotion, recreational resource management

## Abstract

The purpose of the study is to identify a set of key indicators with weightings for health tourism destinations by using an advanced analytic hierarchy process (AHP) method, derived from the official, academic, and professional opinions of the experts. The AHP method allocated weightings to the evaluation criteria selected by the fifteen experts. After expert evaluations were conducted, the three dimensions and eleven sub-dimensions of the initial health tourism destination were obtained as follows: (1) special demands and indications—medical care, health promotion, and tourism and leisure; (2) natural environment—climate, air, water, and light; (3) leisure activities and general demands—sports, therapeutic activities, interactions with animals and plants, and diet. The results revealed that the dimensions of special demands and indications were given the most attention and that the sub-dimensions of sports promotion were the highest ranked by expert groups. The official and academic opinions suggested that health tourism destinations should focus on special demands and indications, while professionals tended to consider the natural environment as a primary concern. In particular, they considered that good air quality can help people release pressure, relax, activate lymphocytes, improve immune function, and enhance disease immunity. The health tourism destination index can contribute to the overall strategic planning process by identifying improvements in activities and enhancing competitiveness in health tourism management by using benchmarking to further improve tourists’ experience and satisfaction.

## 1. Introduction

The Healthy China 2030 initiative promotes the integration of health-related industries in order to develop standards and accelerate the growth of health tourism [1]. Many health tourism industries are strong drivers of economy and society, and have been identified as the economic pillars of developed countries. Currently, China’s health tourism industry is in its infancy because the integration of industries is insufficient, no unified planning at the policy level is available, and a complete industry has yet to be formed. Although exploration and innovation initiatives have been conducted, such tasks are insufficient as a basis for promulgating policies to standardize and guide the development of the health tourism industry. If we wish to make the concept of health deeply rooted in people’s lives, health must be prioritized, diversified health tourism needs of the public must be met, and health tourism destinations must be accessible to people [2].

The concept of health tourism originated from the definition by the International Union of Official Travel Organizations (1973), the predecessor of the United Nations World Tourism Organization, defined as “providing health facilities in rural areas with natural resources, especially hot spring areas and unique climate areas.” Travel medicine involves traveling to different countries. It is related to disease prevention, injury, immunology, infectious diseases, and vaccination. Medical travel, such as for health inspections and plastic surgery, involves medical treatment, rehabilitation, and self-care. In wellness tourism, tourists travel to rural areas with abundant natural resources, such as forest resorts, for disease prevention and treatment [3]. Leisure environments are restorative and revitalizing. Health tourism provides theses environments to restore tourists’ health.

People living in urban cities experience environmental stress, such as from noise, congestion, air pollution, pressure from interpersonal relations, and pressure from a fast-paced lifestyle; these adversely affect human health. To solve these problems, parks, gardens, and amusement parks are established in cities. Moreover, people leave cities to relax, improve their physical fitness, and engage in social activities, because natural environmental factors, including sound, temperature, humidity, atmospheric pressure, air, light, and lack of crowds, have many health promotion effects that cannot be replicated in urban cities [4]. Tourists can enjoy the natural environment through health tourism, which provides entertainment, health benefits, and health education on topics such as environmental hygiene, accident prevention, and allergy prevention [5,6]. Postoperative patients can be rehabilitated by performing normal physiological functions to achieve desired effects in nonmedical settings. Health tourism provides tourists with different degrees of perceptual stimulation through new environmental experiences, in order to elicit a positive effect on their health and behavior, increasing wellbeing and preventing diseases.

Although health tourism has received attention from the public and private organizations, the increasing demand for health destinations indicates that tourism scholars must pay attention to health tourism. Previous tourism destination studies have focused on economic models [7], health environment assessments [8], destination satisfaction [9,10], medical tourism destinations [11], demand for destinations [12], restaurants and destinations [13], destination safety [14], and destination competitiveness [15,16]. However, few studies have determined key indicators and parameter that can be used by the health tourism industry to evaluate health tourism destinations. These methods used for researching health tourism destinations are monotonous and conventional; scientific and empirical methods are lacking. Therefore, establishing a scientific method for studying the standards of health tourism destinations is essential. The aim of this study was to develop a health tourism destination evaluation system, establish indicators by using an analytic hierarchy process (AHP) approach, and validate such indicators using field tests.

This study makes two main contributions. First, understanding the choices and behaviors of health tourists toward destinations is crucial. This study shows that regular and systematic analysis of evaluated results can assist those in the health tourism industry and national and regional destination management organizations in effectively understanding the gaps between the services that health tourism provides and the requirements of health tourists. Second, the health tourism destination index can contribute to the overall strategic planning process by identifying improvements in activities and can enhance competitiveness in health tourism management using benchmarking to further improve tourists’ experience and satisfaction.

## 2. Background Conceptions

### 2.1. Demarcation of Health Tourism

**Figure 1:**
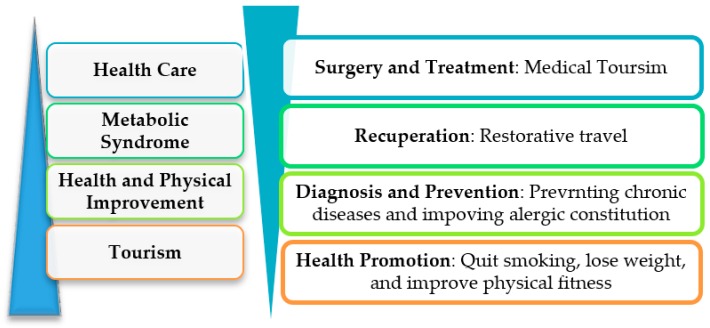
Categories of health tourism, including both leisure and medical aspects.

According to tourists’ health status, health tourists can be divided into those seeking leisure-oriented tourism, health-promoting tourism, and medicine-oriented tourism. Leisure-oriented tourists seek risk-taking activities and new experiences during travel, whereas medicine-oriented tourists prefer to have their diseases treated and their health restored [17]. Compared with Mueller and Kaufmann’s study in 2001, which classified health tourism from tourists’ perspectives [17], the Japanese Institute of Tourism Research (JITR) divided health tourists into four types based on experts’ perspectives, according to their leisure and medical activities: (1) Tourism—tourists desire sightseeing, learning, and tourism experiences based on health; (2) health and physical improvement—tourists want to improve exercise habits, reduce stress, enhance physical strength, and go sightseeing; (3) metabolic syndrome—tourists want to combat metabolic syndrome, for example through playing sports and learning; (4) healthcare—tourists demand to travel in a healthy manner and to pay for diet and mental health. Health tourism can be divided into five types, namely surgery and treatment, recuperation, diagnosis and prevention of diseases, health promotion, and leisure, as illustrated in Figure 1 [18]. Surgery and treatment pays most attention to medical factors and the least attention to leisure factors, which is the opposite of leisure tourism. Health tourism covers types 2 to 5. Indeed, previous studies on health tourism have been explored regarding in-depth classifications; however, a pairwise comparison approach is still uncommon.

### 2.2. Measurement of the Health Tourism Destination Index

An index provides simple numbers for a complex phenomenon and allows a relatively objective comparison using quantitative and qualitative methods [19]. The quality of a health tourism destination is determined based on the ability of medical personnel and those providing therapeutic services to harness the natural environment for resources and services such as mineral springs, mud, carbon, seawater, and climate therapies. In addition to preserving culture and providing high-quality services and social communication, the environment and landscape should be utilized to allow tourists to have a positive and open experience [20,21].

**Figure 2:**
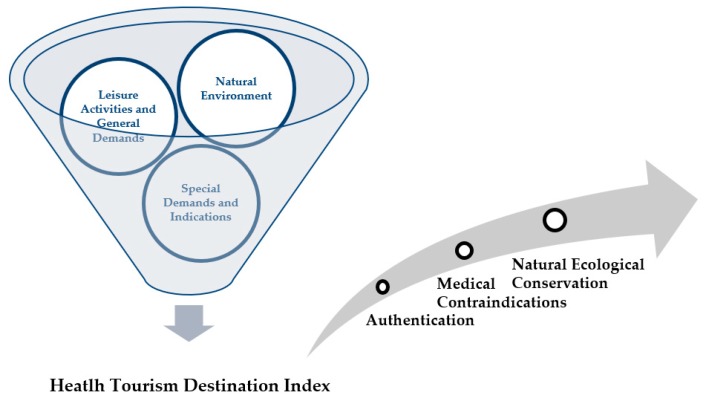
Development of a health tourism destination index.

Health tourists desire environments that are optimally stimulating. When tourists are engaged in leisure activities and health tourism, if they are in an optimal environment, they can avoid negative environmental stimuli so as to feel secluded and comfortable; this can lead to health promotion. Furthermore, if tourists are in a stimulating environment, they can strengthen their physiological and psychological functions to adapt to such an environment. In the Health Tourism Destination Index of Germany, the four indicators of soil, sea water, climate, and alternative therapy are divided according to whether they are part of therapeutic and general tourism sites. These four aspects must be satisfied for a site to be considered to be therapeutic [6]. The combination of soil and spring water should provide recuperation, as proven by a physician’s investigation. Alternative therapies include exercise and therapeutic gymnastics, which can relieve tension. Furthermore, various dietary therapies are provided according to different indications to improve the physical and mental health of convalescents [22]. Therefore, the identification of healthy tourism destinations should be based on five criteria: recuperation, climate and air quality, general and special requirements, indications and contraindications for environmental protection, and medical identification [23,24,25,26,27,28,29,30,31,32]. The study developed an applicable index for healthy tourism destinations comprising three dimensions, namely the natural environment, leisure activities and general demands, and special demands and alternative therapy indications. Through this approach, relevant standards were established. By using these three dimensions, we aimed to develop certification for healthy tourism destinations, as shown in Figure 2. Natural environment, leisure activities and general demands, and special demands and indications are described in the following section.

### 2.3. Activities in Health Tourism Destinations

Activities in health tourism destinations are motivated by medical treatment and health promotion, and can be divided into acute medical treatment, chronic medical treatment, health promotion, and leisure [19,33]: (1) Acute medical treatment—accepting emergency medical treatment in medical institutions is necessary (health tourism is not applicable); (2) medical treatment—there are many medical facilities and services to provide benefits for those with chronic conditions, such as joint pain, traumatic sequelae, frozen shoulders, neuralgia, diabetes, gout, ectopic dermatitis, autonomic nervous disorder, and motor neuron injuries; (3) health promotion—this can be divided into two subtypes, disease prevention (prevention of motor dysfunction caused by aging and sleep disorders, improvement of allergic constitution, and increase in anti-pressure ability) and health enhancement (enhancement of cardiopulmonary function, muscle strength, and muscle endurance); (4) leisure—this type of health tourism focuses on entertainment, health maintenance, self-development for relaxation, the provision of psychological relief, skin condition improvement, and other cosmetic effects. This type of activity helps people to feel refreshed and enriched; furthermore, it improves their motivation and sports performance. The above methods have numerous purposes, including the cultivation of sport habits, the adjustment of dietary habits, and inspiring interaction between tourists and animals and plants. According to the differences in health tourism motivations, there are different types of health tourism, such as for medical care, recuperation, the prevention of chronic diseases, the improvement of allergic constitution, smoking cessation, weight loss, improving physical fitness, and expanding the mind. Special requirements and indications can be considered in relation to the provision of health tourism products, such as hot spring spas, cosmetology, health examinations, mud therapy, and organic food provision [18,19,34].

#### 2.3.1. Indicators of Leisure Activities and General Demand

Interpersonal interaction, cultural and leisure activities, and recreational activities are the main factors that attract engagement in these types of tourism. In addition to health benefits, health tourism destinations must provide entertainment and activities that strongly motivate tourists to go to such destinations. The characteristics of health tourism destinations are different; therefore, leisure activities are different, such as sports, farming, music, massage, diet, and spiritual exploration. General demand refers to tourists’ motivations for basic services in health tourist destinations, including accommodation, catering, and transportation [21,29,30,31].

#### 2.3.2. Indicators of Special Demands and Indications

Health tourism destinations can meet requirements according to different indications; they can provide health-related entertainment, an environment of relaxation, health education, and personal nutrition improvements. Health tourism has benefits related to environmental hygiene as well as accident and allergy prevention. Special attention should be paid to the effects of the environment on human health and tourists’ experiences. Health tourists must be treated. To restore normal physiological function, rehabilitation processes such as functional therapy, physiotherapy, and psychotherapy can be conducted in medical or nonmedical environments in health tourism; to achieve such goals, these destinations must provide a safe environment and high-quality facilities.

## 3. Methodology

It is necessary to refer to the criteria of the tourism industry to identify health tourism destinations and to develop a related index. This study aimed to develop certification standards for tourist destinations by considering aspects such as environmental and ecological protection and the sustainable development of health tourism. Based on the concept of preventive medicine, the study evaluated the feasibility and appropriateness of a health tourism destination index and eliminated factors unsuitable for related projects. After expert evaluations were conducted, three dimensions and eleven sub-dimensions of the initial health tourism destination were obtained as follows: (1) special demands and indications—medical care, health promotion, and tourism and leisure; (2) natural environment—climate, air, water, and light; (3) leisure activities and general demands—sports, therapeutic activities, interactions with animals and plants, and diet.

This study confirmed the importance of each index by using the AHP. The AHP is a multilevel analysis method used to deconstruct a problem at a dendritic structure level, to establish a class structure level with a mutual influence, and to facilitate more accurate decision-making for complex issues [16]. Two items at each level with different measurements were compared and the comparative matrixes were paired, which were established to calculate the number of featured vectors and to represent the priority of crucial elements at structural levels [35]. The featured value was then calculated, which formed an evaluative basis for judging the level of consistency and the extent of influence on each comparative matrix [36]. During problem evaluation, experts considered the weights of the solutions as a reference for decision making [37]. This study explored and adjusted the experts’ judgment of the index weights of health tourism destinations. Because the AHP involves the use of a questionnaire with a special structure, the selection of respondents was limited to experts with objective knowledge of the questionnaire. The criteria were chosen by scholars, officers, and professionals in the fields of health and tourism or personnel from relevant government or industrial units, social tourism bodies, and healthcare institutions. Three types of experts were interviewed individually, namely five teaching and scientific research professors of universities; five officials from current or former government tourism-related institutions who are responsible for tourism-related business or job distribution and integration; and five professionals from the tourism, health, and insurance industries who are senior employees and project managers, bringing the total number of experts to 15. After questionnaire data were collected using the AHP analysis website “Expert Choice Comparison”, a comparison matrix was established to test the consistency of the dimensions (consistency ratio (CR): <0.1). Then, the weights of the dimensions were determined. The AHP was conducted to avoid decision-making fatigue caused by the presence of too many weight comparisons among respondents. Each dimension has a maximum of seven questionnaires [36]. The four steps for the AHP were as follows: (1) finalization of the evaluation criteria system; (2) questionnaire evaluation; (3) allocation of weighting and consistency clarification; (4) calculation of the weighted values for each evaluation [36].

## 4. Results and Discussion

The experts assessed AHP questionnaires related to health tourism destinations. There were three sets of dimensions and eleven sub-dimensions for consistency ratio determination. Because the CR was <0.1, consistency was considered. The results of consistency determination revealed that all eleven questionnaires were valid and applicable and that the weights of the health tourism destination index were as follows: 38.01% for special demands and indications, 32.24% for leisure activities and general demands, and 29.75% for natural environment factors. The top six assessments of sub-dimensions were health promotion (14.84%), diet (13.86%), sightseeing and leisure (12.64%), air (11.52%), exercise (10.76%), and chronic medical treatment (10.53%) (Table 1).

The results indicated that the dimensions of special demands and indications were given the most attention and that the sub-dimensions of sports promotion, sightseeing and leisure, and chronic medical treatment were highly ranked. The results were consistent with those of previous studies that revealed that health tourism destinations should provide high-quality sightseeing and healthcare services along with health promotion [4,8]. Therefore, health tourism destinations should provide more healthy leisure products. Health tourists can enhance their immunity to diseases through purposeful physical activities by releasing their mind and eliminating the accumulated pressure of daily life. Moreover, health tourism destinations should provide services related to disease treatment and recuperation. By using the advantages of the local environment and resources, healthy tourism destinations can provide dynamic leisure activities and combine medical and health resources in order to establish healthcare tourism products. Such an approach can help tourists have positive experiences and recuperate successfully.

For the dimensions of leisure activities and general demands, the sub-dimensions of sports and diet were favored by experts. German and Japanese studies on health tourism destinations have indicated that walking and a healthy diet can reduce blood pressure [38]. Traditional martial arts, qigong, and the concept of eating conform to the “four seasons” approach and enhance the body’s adaptability and immunity [39,40]. Chinese medicine tourism is beneficial for the treatment of chronic diseases, which is achieved through various changes in exercise and diet [14]. Therefore, health tourism destinations should highlight the health benefits of dietary nutrition, pay attention to scientific nutritional dietary concepts, promote healthy cooking methods, and combine Chinese martial arts and other activities such as guided during mountain treks and sea exploration. Health tourism destinations near mountainous areas can offer directional activities, cross-country running, rock climbing, grass skiing, mountain biking, hiking, and climbing, whereas health tourism destinations near the ocean can offer activities ball sports, jogging, waterfront walking, swimming, and boating.

This study emphasized the importance of natural environmental dimensions, especially the sub-dimension of air. Investigations of health tourism resources should consider the importance of air-related recuperation. Negative ion air environments provide effective disease recuperation and healthcare effects for tourists, as well as positive effects related to anti-aging, sedation, sleep, blood pressure regulation, appetite enhancement, lung function, and immunity. In an environment with clean air, recreational projects such as walking and camping, Buddhism-related health preservation, and tea-related healthcare can be conducted, and negative ion breathing areas, recuperation rooms, and mountain and water bars can be developed [6,20,40]. Health tourism destinations should vigorously publicize the efficacy of negative air ions and expand the ecological and health tourism market.

The officers and scholars rated special demands and indications as the dimensions with the highest weighting (45.87% and 61.61%, respectively), whereas the professionals rated the natural environment dimension the highest (45.13%). Overall, experts rated dimensions of leisure activities and general demands the second highest (28.89%, 24.30%, and 34.28%). The officers and scholars rated the natural environment dimension the lowest (25.24% and 14.09%), whereas the professionals gave the natural environment a rating of 20.59%. The difference in the weighting of health tourism destinations among different expert groups can be determined by using median absolute deviation (MAD), which had high consistency with the opinions of experts. Conversely, a high MAD value indicated divergence with the opinions of experts. The sub-dimensions animals and plants and diet exhibited the highest consistency (MAD = 0.44), which are ranked between 10th and 11th and 3rd and 4th, respectively. The climate sub-dimension had an importance ranking between 5th and 8th (MAD = 1.11). The light sub-dimension had an importance ranking between 8th and 11th (MAD = 1.33). The sub-dimensions of sport and health promotion had importance rankings between 2nd and 7th and 1st and 6th, respectively (MAD = 1.78). The sub-dimensions of water and therapeutic activities had importance rankings between 4th and 9th and 1st and 6th, respectively (MAD = 2). The sub-dimensions of tourism and leisure had importance ranking between 1st and 7th (MAD = 2.22). The air sub-dimension had an importance ranking between 1st and 7th (MAD = 2.44). The medical care sub-dimension had an importance ranking between 2nd and 9th (MAD = 3.11) (Table 2). The groups of officers and scholars tended to agree that health tourism destinations should focus on special requirements and indications. This result was congruent with Marc and Renee’s study [19]. They considered the sub-dimensions of tourism, health promotion, and medical care to be crucial to developing successful health tourism destinations. Health tourism destinations should be health-oriented and should attract tourists by using the unique characteristics of health resources. Professionals tended to consider the natural environment as a primary concern. In particular, they considered that good air quality can help people release pressure, relax, activate lymphocytes, improve immune function, and enhance disease immunity. For instance, many forest hospitals in Germany are established in locations with good air quality to help people absorb pythoncidere [20,41].

Moreover, all expert groups agreed that the second most important sub-dimensions are those of leisure and general demands, especially diet and activities related to animals and plants. Restaurants are important amenities for tourism destinations [13]. In the heath tourism destinations, not only can healthy diets using local ingredients provide changes to tourists’ taste buds, but using local meat and plants can comfort tourists’ souls. Health tourism destinations should provide physical, mental, and spiritual experiences for tourists.

## 5. Conclusions and Future Suggestions

From the perspective of health tourism, and based on a literature review, this study developed a health tourism destination index with three dimensions and eleven sub-dimensions. Expert groups composed of specialist researchers and personnel from industries with substantial experience in health tourism were selected to conduct AHP. The conclusions and recommendations drawn from the results are as follows.

### 5.1. Establishing the Reliability of the Health Tourism Destination Index

Different expert groups provided various weights for the index of health tourism destinations in the order of special demands and indications, leisure activities and general demands, and the natural environment. The order of sub-dimensions was health promotion, diet, sightseeing and leisure, air, exercise, and chronic medical treatment. The weights of the indicators obtained by the experts for evaluating tourism destinations were not focused on a single indicator, the eleven sub-dimensions selected in this study are suitable for evaluating the health tourism industry. Thus, it is suggested to select several health tourism destinations for empirical research. To evaluate the effectiveness of health tourism, we used actual participation analysis and a questionnaire to understand the effects of different aspects (sightseeing, health promotion, metabolic syndrome, and healthcare). Finally, the results of questionnaire survey and participatory observation were revealed. The health tourism destination index was applied to determine the actual effect of health tourism in order to provide suggestions to suit local conditions.

### 5.2. Strengthening Regional Advantages Using the Health Tourism Destination Index

Health tourism covers five categories: medical sightseeing, convalescence tourism, diagnosis and prevention, health promotion, and leisure. Operations and treatments are performed according to different purposes, such as convalescence travel after an operation or treatment, the prevention of chronic diseases, improvement of physical fitness (e.g., to improve mental health, stop smoking, and for weight control), and related parameters, including self-development, self-exploration, and happiness. The concept of health tourism is based on the relationship between the natural environment and humans. When traveling to a health tourism destination, tourists respond to the environment by feeling comfortable or stimulated, without direct medical treatment or healing.

The development of health tourism destinations should start by focusing on medical treatment, health management, nursing, and recuperation. The industry relies on local ecologies, communities, and natural resources; the integration of health-tourism-related industries; the improvement of industry management systems and service quality; and the implementation of preferential policies related to tax and investment. Further, health tourism could encourage investment related to social capital. Under these circumstances, tourists can be provided with a complete travel itinerary, especially those with chronic medical conditions. Sightseeing and other leisure activities, as well as a healthy diet, can improve their health. A region is considered a health tourism destination when people can live there healthily and happily and when the region develops a health tourism industry; this can provide local economic development. Residents can practice and share their healthy life habits to educate tourists on the idea of healthy tourism, whereas tourists can learn how to improve their daily life and promote their health. Health tourism could become a popular alternative therapy in the future, and health tourism destinations could become sustainable.

This was an empirical study conducted to establish the key dimensions and sub-dimensions for a health tourism destination index from the supply (health and tourism) perspective; further conclusive explanatory research is required to test the developed index and weightings. The key dimensions of health tourism destinations developed in this research are based on the expert opinions provided in the Chinese cultural context. Therefore, studies should be conducted with experts from other countries who can share different perspectives, leading to the attainment of crucial comparative results. This, in turn, could contribute to the development of a more comprehensive measurement technique, with a focus on the interaction between cultures and societies. Moreover, many communities in health tourism destinations express a desire to develop a health tourism program [20]. Future studies could investigate new cultural, social, and regional factors to test the index cross-culturally, in order to further establish validity.

## Figures and Tables

**Table 1:** Overall assessment of health tourism destinations.

Dimensions Level 1	Level 1 Weight *	Sub-Dimensions Level 2	Level 2 Weight *	Overall Weight *	Sort
Special Demands and Indications	38.01	Medical care	27.69	10.53	6
		Health promotion	39.04	14.84	1
		Tourism and leisure	33.26	12.64	3
Natural Environment	29.75	Climate	26.34	7.84	7
		Air	38.72	11.52	4
		Water	23.49	6.99	8
		Light	11.46	3.41	10
Leisure Activates and General Demands	32.24	Sport	33.38	10.76	5
		Therapeutic activities	14.93	4.81	9
		Animals and Plants	8.40	2.71	11
		Diet	43.30	13.96	2
Total	100%		100%	100%	

* Weights (%).

**Table 2:** Expert group assessments of health tourism destinations.

Dimensions Level 1	Sub-Dimensions Level 2	Officers Level 1 Weight	Officers Level 2 Weight	Officers Overall Weight	Sort	Scholars Level 1 Weight	Scholars Level 2 Weight	Scholars Overall Weight	Sort	Professionals Level 1 Weight *	Professionals Level 2 Weight *	Professionals Overall Weight *	Sort	MAD
Special Demands and Indications	Medical care	45.87	28.89	13.25	2	61.61	36.07	22.22	2	20.59	21.10	4.34	9	3.11
	Health promotion		22.50	10.32	4		37.93	23.37	1		47.30	9.74	6	1.78
	Tourism and leisure		48.61	22.29	1		26.00	16.02	3		31.59	6.50	7	2.22
Natural Environment	Climate	25.24	31.12	7.86	6	14.09	27.76	3.91	8	45.13	22.86	10.32	5	1.11
	Air		42.18	7.77	7		42.18	5.95	6		39.56	17.85	1	2.44
	Water		17.63	6.73	8		17.63	2.48	9		27.44	12.38	4	2
	Light		12.43	2.88	11		12.43	1.75	11		10.14	4.58	8	1.33
Leisure Activities and General Demands	Sport	28.89	28.39	8.2	5	24.30	20.53	4.99	7	34.28	45.21	15.5	2	1.78
	Therapeutic activities		16.47	4.76	9		29.48	7.16	5		7.83	2.69	10	2
	Animals and Plants		13.14	3.8	10		7.73	1.88	10		6.54	2.24	11	0.44
	Diet		42.00	12.13	3		42.27	10.27	4		40.42	13.86	3	0.44
Total		100%	100%	100%		100%	100%	100%		100%	100%	100%		

* Weights (%). MAD: median absolute deviation.
